# An Exploratory Study of Inflammatory Bowel Disease and the Psychosocial Factors Affecting Health-Related Quality of Life

**DOI:** 10.3390/medsci7020018

**Published:** 2019-01-25

**Authors:** Nirmala Sarwan, Ricardo Jurawan, Rudrunath Singh, Vijay Kumar Chattu

**Affiliations:** 1 Department of Gastroenterology, Port of Spain General Hospital, North West Regional Health Authority, Port of Spain, Trinidad and Tobago; nirmalasarwan88@gmail.com (N.S.); ricardo.jurawan@gmail.com (R.J.); 2 Department of Paraclinical Sciences, Faculty of Medical Sciences, The University of the West Indies, St. Augustine, Trinidad and Tobago; rudrunath.singh@gmail.com

**Keywords:** inflammatory bowel diseases, ulcerative colitis, Crohn’s disease, health-related quality of life, psychosocial factors, illness perception

## Abstract

Health-related quality of life (HRQoL) is a multidimensional concept that assesses an individual’s personal satisfaction with his/her daily life while coping with a medical condition and/or its consequent therapy. This study aims to determine the psychosocial needs most commonly affected among inflammatory bowel disease (IBD) patients. Psychosocial needs which were pertinent to the IBD community were assessed using a questionnaire designed by the gastrointestinal team at the Port of Spain General Hospital, Trinidad after getting ethical approval. The chi-squared test was used to assess for statistically significant associations. Of the total 115 participants who completed the survey, a majority of 73 (63%) were females and 70 (61%) were in the 18 to 40-year age group. A majority of 66 (57%) were diagnosed with ulcerative colitis and 66 (57%) were on non-biologic drug therapy. Diet was the need most prevailingly affected, with 87% of participants indicating such. Ability to maintain good hygiene was the need least affected, with 32% of participants identifying concern. We found a significant association between participant’s illness perception (IP) and each of the ten psychosocial variables. The participants’ IP was in turn strongly associated with the ability to cope with the illness while not being associated with the demographic or clinical details of the participants. The results can be used to improve the quality of care delivered to patients living with IBD.

## 1. Introduction

Health-related quality of life (HRQoL) is a multidimensional concept that assesses an individual’s personal satisfaction with his/her daily life while coping with a medical condition and/or its consequent therapy. This is measured through the person’s state of physical, mental, emotional and social well-being [1]. HRQoL is a principal determinant of outcome in chronic illnesse, including inflammatory bowel diseases (IBD). Inflammatory bowel disease encompasses Crohn’s disease (CD) and ulcerative colitis (UC), and is characterized by chronic inflammation of the gastrointestinal (GI) tract. The cause of IBD is inconclusive but it appears to be the result of an interplay between genetic and immune factors [2]. IBD is a global disease, and there are several studies throughout the American, European and Asian continents identifying its prevalence. In North America and Europe, over 1.5 million and 2 million people suffer from the disease, respectively [3]. Despite these numbers, the public awareness of IBD as a chronic disease is relatively limited. Symptoms of IBD are mainly in the form of abdominal pain, bloody diarrhea, and urgency of bowel movements which may result in fecal incontinence. Frequently associated symptoms outside of the digestive tract include fatigue, joint pains, and weight loss. Chronic inflammation of the bowel may give rise to malnutrition; anemia; bowel fistulae, perforation or obstruction; and colonic cancer.

The disease often has a negative effect on the patient’s emotional and social life, which are not always visually apparent. Common fears among patients include unanticipated flares, loss of bowel control, producing offensive odors, shortened life expectancy, inability to lead normal working and family lives, risk of colon cancer, the need for surgery and the need for an ostomy bag [4]. Furthermore, the uncertainty of the disease course makes it challenging for patients to make decisions regarding their work and daily activities, which may result in unemployment or social isolation [5]. As a result, however not limited to these factors, a significant number of patients with IBD believe that their quality of life (QoL) is adversely affected by their disease [6]. This has been corroborated by studies which indicate reduced QoL compared with an age-matched and sex-matched population [7]. 

Despite the large number of studies which highlight the negative effect of IBD on QoL, there are only few studies which explore how IBD impacts of the psychosocial needs of patients. This is an important area to explore due to the chronicity of the illness, its high incidence in a younger population, the often-embarrassing nature of its presentation and the limited public awareness of the disease. These factors combined present daily challenges to sufferers, many of whom are in the critical years of social and professional development. This study aims to explore which psychosocial needs are affected among IBD patients and to determine whether these needs differ by the age, gender, IBD subgroup or treatment modality of patients. It also aims to document how the psychosocial health aspects of patients are linked to their illness perception (IP). IP is defined as the beliefs a patient holds about his/her health and several studies have identified IP as a strong predictor of psychological distress and poorer QoL even after accounting for disease activity [8,9].

## 2. Methodology

### 2.1. Sample 

Data for this cross-sectional study was collected by means of a questionnaire designed by the authors after getting the approval from the Research Ethics Committee. The questionnaire was distributed at the Gastrointestinal Unit at the Port of Spain General Hospital. Patients who met the inclusion criteria (a tissue diagnosis of UC or CD; and of age 18 years or older), were invited to fill the questionnaire in the waiting room before their regular physician appointment. Participation in the survey was optional, with no repercussions to those who declined. All questions were in close-ended format, with an additional free comment section at the end. Data collection took place between November 2017 and March 2018. The questionnaire included sections on demographic details, clinical details, and quality of life assessment.

### 2.2. Quality of Life Assessment

A unique set of questions were formulated to assess the psychosocial domain of HRQoL. We identified 10 psychosocial needs which were pertinent to the IBD population. They were: (1) dietary restrictions, (2) difficulty maintaining proper hygiene, (3) episodes of incontinence, (4) deflated self-esteem, (5) feelings of unattractiveness, (6) strained relationships with others, (7) fear of intimacy, (8) limitations to leisure and hobbies, (9) lack of clear-mindedness and (10) loss of independence. Participants were also asked to rate their illness perception, to identify whether they had adequate social support and to identify whether they had difficulty in coping with their illness.

### 2.3. Statistical Analysis

The participants were enrolled in the study as per the inclusion criteria and we collected the required information through the questionnaires. In the next phase the information gathered was coded and entered in an Excel sheet. The complete dataset was processed and analyzed using SPSS (Version 25.0). Initial descriptive analysis included the exploration of clinical and demographic data using primarily frequencies, percentages, quartiles and ranges. Pearson’s chi-squared tests of independence (including Fisher’s Exact Test for small sample frequencies) were conducted for significant associations between psychosocial parameters and several independent variables, such as age, gender and IBD subgroups which include UC and CD. Further analysis using binary and multinomial logistic regression explored the probabilities of illness perception against selected variables arising from the chi-squared tests. A 0.05 level of significance was used for statistical tests.

## 3. Results

A total of 115 patients returned completed questionnaires. Of this study sample, 73 (63%) were female and 42 (37%) were male. Sixty-six (57%) participants had been diagnosed with UC and 49 (43%) with CD. The age of participants ranged from 19 to 82 years, with a median age of 37 years (interquartile range = 29 to 50 years). Sixty-one percent (61%) of patients fell into the 18 to 40-year age group. Disease duration of participants ranged from 1 to 42 years, with a median of 7 years (interquartile range = 5 to 14 years). Sixty-six patients were taking non-biologic drugs while 31 were taking biologic drugs. Further clinical details are shown in Table 1.

For each of the ten psychosocial needs identified, participants were asked to respond ‘yes’ or ‘no’ if they felt the need was affected by their illness. The findings are shown in Table 2. The top three needs which were affected, in descending order of percentages, were diet (87%), clear-mindedness (61%) and leisure/hobbies (57%). The chi-squared test for association between the psychosocial needs and the participants’ age, gender, IBD subgroup and treatment modality generated *p*-values of 0.7314, 0.9986, 0.9744 and 0.987, respectively.

The participants’ IP was assessed on a five-point Likert scale. They were also asked about their ability to cope with their illness daily. The results are displayed in Table 3. In total, 64 (56%) participants indicated that they had difficulty in coping with their illness, while 51 (44%) did not. All participants who perceived their health as either very bad or bad also had difficulty in coping with their illness. For those who perceived their health as reasonable, 65% still had difficulty in coping, while only 19% of those who reported good health continued to have difficulty in coping. All participants who reported very good health also reported no difficulty in coping with illness. Statistical analysis for an association between illness perception and ability to cope with the illness generated a *p* value of <0.000.

Lastly, participants were asked about the adequacy of social support that they received. Ninety-one participants (79%) indicated that they did have adequate support, while 24 (21%) did not.

Bivariate analysis was done to evaluate for a relationship between the participant’s IP and demographic and clinical variables. Same was done for the relationship between IP and each of the ten psychosocial variables. No demographic or clinical variables were found to be associated with the participant’s IP. However, impairment of all ten psychosocial needs was linked to the participant’s IP. Further characterization by odds ratio has shown that participants who have a perception of poor health were less likely to think that their psychosocial needs were affected. The results are shown in Table 4 and Table 5.

## 4. Discussion

This study aims to determine the psychosocial needs most commonly affected among IBD patients and whether impairment in these needs differs by age, gender, IBD subgroup or treatment modality. Lastly it evaluates for an association between psychosocial needs and the participants’ IP. We found that diet was the need most prevailingly affected, with 87% of participants indicating such while ability to maintain good hygiene was the need least affected, with 32% of participants identifying concern. Our data also showed that there was no significant association between psychosocial needs being affected and the participants’ age, gender, IBD subgroup or treatment modality. Alternatively, we found that the individual associations between IP and each of the ten psychosocial variables were significant. In particular, the odds of a participant’s belief that their psychosocial needs are affected decreases as they move from poor IP to good IP. However, the logistic regression analysis indicated that only hygiene and attractiveness were together significantly affected by the participants’ IP.

Numerous studies corroborate the undervalued role of diet in the etiopathogenesis of IBD [10]. According to Zallot et al. (2013), as many as 70% of IBD patients are known to employ restriction diets during remission in order to avoid exacerbation of the disease, which affects both their social and family life [11]. This suggests that even with quiescent disease activity, diet remained a concern among patients. In this regard, Principi et al. (2018) has shown that IBD patients make dietary self-restrictions in fibers intake even after achieving remission [12]. Hou et al. (2014) reviewed patient-targeted dietary information for IBD from structured Internet searches and popular defined diets [13]. They found that patients have strong beliefs about the role of diet in the cause of IBD and in exacerbating or alleviating ongoing symptoms from IBD. However, patient-targeted dietary recommendations were highly conflicting. Data that altering diet can change the natural history of IBD are scarce, and evidence-based dietary guidelines for patients with IBD are lacking. Patients, therefore, seek nonmedical resources for dietary guidance, such as patient support groups and unverified sources on the Internet.

Clear-mindedness was the second most affected need, with 61% of participants in our study being affected. One participant captured its critical implications by stating that: *‘Crohn’s disease has affected every aspect of my life. The unpredictability of Crohn’s disease is the scariest part. Dropping out of university due to illness has caused me to fear even attempting to pursue a career in the field I love because I don’t know how bad the next flare will be. Going to sleep every night not knowing what the next morning will be like is mentally exhausting’.*

Personal relationships are important factors in HRQoL. In our study 54%, 47% and 51% of participants had impaired self-esteem, attractiveness and intimacy respectively. Intimacy in particular was most affected in the 18- to 40-year age group. Knowles et al. (2013) have found that illness perceptions had a significant positive correlation with anxiety, body image and self-consciousness during intimacy, which in turn affected family functioning [14].

Forty-five percent of participants in our study indicated that they had concerns with bowel incontinence. Norton et al. (2013) examined the association between the degree to which the subjects worried about possibility of a bowel accident and the actual reported frequency of incontinence [15]. They found that those with more frequent incontinence were more likely to think about a bowel accident, while two-thirds of people reporting that they never experienced incontinence, reported thinking about it, even if only rarely. Of note, ability to meet hygienic needs was the factor least affected among our cohort and did not correlate with concerns regarding bowel incontinence.

We found there was no significant association between psychosocial needs being affected and the participants’ age, gender, IBD subgroup or treatment modality. Some existing studies support an association between these variables and QoL [7,16,17,18], while others do not [19,20,21,22,23,24,25,26,27,28,29]. The most surprising of these findings is that of treatment modality. Principi et al. (2015) has demonstrated that biologic treatment may strongly improve QoL, especially when it is administered using shortened 1-h protocols [30]. Similarly, Holdam et al. (2017) has found that treatment with biological therapy leads to a statistically and clinically significant improvement in bowel symptoms, interference in daily life, worry, and general well-being. After six months of treatment, bowel symptoms and interference in daily life were similar to patients without disease activity [31].

Our study also failed to find a significant association between social support and IP. However, social support has been extensively studied and is thought to influence HRQoL in IBD patients [32,33,34]. The disease’s impact is often not readily apparent, leading to a lack of understanding from others concerning the presence and graveness of the condition [35]. Two participants in our study alluded to this by stating that: *‘It is challenging to live on your own with this disease and to be considered a burden at times, especially by your family.’* and *‘This illness makes you uncertain. Some mornings are good, then some may be bad. Most employers do not understand. It’s not like you want to be late for work or not attend. Some people cannot comprehend this illness.’*

The primary limitation of our study was the infeasibility for the disease burden of all participants to be assessed at the time of the study. This limitation did not allow for comparison of psychosocial health between groups with measurable disease activity and those in remission. Previous studies which have explored the disease-specific QoL of IBD patients such as those by Turnball et al. (1995) and Graff et al. (2006) showed that those with active disease had poorer QoL relative to those with inactive disease [36,37]. In contrast, Lix et al. (2008) have found that the subjective experience of ill health associated with IBD does not always correlate with clinical disease activity and the disease continues to impact on the individual’s psychological status even when in remission [38]. Thus, further research should be undertaken to explore the relationship between psychosocial functioning and clinical disease activity.

## 5. Conclusions

Inflammatory bowel disease remains a commonly encountered gastrointestinal disorder. Though a small sample population, this study can be used to bolster further research into innovative ways to identify and deal with unmet psychosocial needs of patients living with IBD. The key strategies can include routine psychological assessment for patients, dietary consultations and healthcare professionals proactively enquiring about issues such as sexual health and bowel incontinence. Evidence-based dietary guidelines for patients with IBD are needed to address the overwhelming concern of patients with diet. Secondly, the organization of awareness campaigns for the general public as well as employers are useful adjuncts to patient support groups.

## Figures and Tables

**Table 1:** Demographic and clinical details of the participants.

Variable	*N*
**Gender**	
Male	42
Female	73
**Inflammatory Bowel Disease (IBD) Subgroup**	
Ulcerative colitis	66
Crohn’s Disease	49
**Age Groups**	
18–30	35
31–40	35
41–50	17
51–60	20
61–70	3
Over 70	5
**Duration of IBD**	
≤5 years	39
6–10 years	34
11–15 years	14
16–20 years	14
21–25 years	5
>25 years	4
Not specified	5
**Treatment Modality**	
None	8
Non-biologic drugs *	66
Biologic drugs **	31
Surgery	19
Partial bowel resection	8
Total colectomy	8
Repair of bowel abscesses and fistulae	4
Ostomy present	5
**Number of IBD Related Hospital Admissions in the Last Year**	
None	76
One	18
Two	8
Three	5
More than three	8

* Non-biologic drugs included oral steroids, 5-aminosalicylic acid, and azathioprine. ** Biologic drugss included infliximab and adalimumab.

**Table 2:** Comparison of psychosocial needs by age, gender, IBD subgroup and treatment modality.

Psychosocial Needs Affected by Illness	Total		Age Group (Years)										Gender				IBD Subgroup				Treatment Modality			
			18–30		31–40		41–50		51–60		Over 60		Female		Male		UC		CD		Biologic Drugs		Non-Biologic Drugs	
	Yes	No	Yes	No	Yes	No	Yes	No	Yes	No	Yes	No	Yes	No	Yes	No	Yes	No	Yes	No	Yes	No	Yes	No
Diet, *n* (%)	100 (87)	15 (13)	34 (97)	01 (03)	33 (94)	02 (06)	15 (88)	02 (12)	13 (65)	07 (35)	05 (63)	03 (37)	63 (86)	10 (14)	37 (88)	05 (12)	55 (83)	11 (17)	45 (92)	04 (08)	30 (97)	01 (03)	56 (85)	10 (15)
Hygiene, *n* (%)	37 (32)	78 (68)	09 (26)	26 (74)	12 (34)	23 (66)	05 (29)	12 (71)	10 (50)	10 (50)	01 (13)	07 (87)	23 (32)	50 (68)	14 (33)	28 (67)	19 (29)	47 (71)	18 (37)	39 (63)	13 (42)	18 (58)	20 (30)	46 (70)
Continence, *n* (%)	52 (45)	63 (55)	23 (66)	12 (34)	20 (57)	15 (43)	06 (35)	11 (65)	03 (15)	17 (85)	00 (00)	08 (100)	34 (47)	39 (53)	18 (43)	24 (57)	30 (45)	36 (55)	22 (45)	27 (55)	17 (55)	14 (45)	28 (42)	38 (58)
Self-esteem, *n* (%)	62 (54)	53 (46)	24 (69)	11 (31)	23 (66)	12 (34)	10 (59)	07 (41)	05 (25)	15 (75)	00 (13)	08 (100)	39 (53)	34 (67)	23 (55)	19 (45)	34 (52)	32 (48)	28 (57)	21 (43)	21 (68)	10 (32)	31 (47)	35 (53)
Attractiveness, *n* (%)	54 (47)	61 (53)	26 (74)	09 (26)	16 (46)	19 (54)	08 (47)	09 (53)	04 (20)	16 (80)	00 (00)	08 (100)	34 (47)	39 (53)	20 (48)	22 (52)	32 (48)	34 (52)	22 (45)	27 (55)	19 (61)	12 (39)	27 (41)	39 (59)
Relationships, *n* (%)	62 (54)	53 (46)	23 (66)	12 (34)	21 (60)	14 (40)	09 (53)	08 (47)	09 (45)	11 (55)	00 (00)	08 (100)	42 (58)	31 (42)	20 (48)	22 (52)	31 (47)	35 (53)	31 (63)	18 (37)	22 (71)	09 (29)	28 (42)	38 (58)
Intimacy, *n* (%)	59 (51)	56 (49)	20 (57)	15 (43)	23 (66)	12 (34)	08 (47)	09 (53)	07 (35)	13 (65)	01 (13)	07 (87)	38 (52)	35 (48)	21 (50)	21 (50)	30 (45)	36 (55)	29 (59)	20 (41)	22 (71)	09 (29)	27 (41)	39 (59)
Leisure, *n* (%)	66 (57)	49 (43)	26 (74)	09 (26)	24 (69)	11 (31)	09 (53)	08 (47)	06 (30)	14 (70)	01 (13)	07 (87)	40 (55)	33 (45)	26 (62)	16 (38)	36 (55)	30 (45)	30 (61)	19 (39)	23 (74)	08 (26)	32 (48)	34 (52)
Clear mindedness, *n* (%)	70 (61)	45 (39)	27 (77)	08 (23)	23 (66)	12 (34)	09 (53)	08 (47)	10 (50)	10 (50)	01 (13)	07 (87)	46 (63)	27 (37)	24 (57)	18 (43)	42 (64)	24 (36)	28 (57)	21 (43)	23 (74)	08 (26)	34 (52)	32 (48)
Independence, *n* (%)	58 (50)	57 (50)	24 (69)	11 (31)	17 (49)	18 (51)	10 (59)	07 (41)	06 (30)	14 (70)	01 (13)	07 (87)	39 (53)	34 (47)	19 (45)	23 (55)	30 (45)	36 (55)	28 (57)	21 (43)	22 (71)	09 (29)	28 (42)	38 (58)

UC: ulcerative colitis, CD: Crohn’s disease.

**Table 3:** Comparison of illness perception and coping capacity of participants.

Participants’ Illness Perception	Ability of the Participant to Cope with the Illness	
	Difficulty in Coping	No Difficulty in Coping
Very Good, *n* (%)	0 (0)	3 (100)
Good, *n* (%)	6 (19)	26 (81)
Reasonable, *n* (%)	41 (65)	22 (35)
Bad, *n* (%)	15 (100)	0 (0)
Very Bad, *n* (%)	2 (100)	0 (0)
Total, *n* (%)	64 (56)	51 (44)

**Table 4:** Bivariate analysis of demographic and clinical variables with participants’ illness perception.

Demographic and Clinical Variables	*p*-Value (Chi-Squared Test of Independence)
Age group	0.179
Gender	0.290
IBD subgroup	0.592
Disease duration	0.898
Treatment modality	0.277
Number of IBD related hospital admissions per year	0.106
Presence of social support	0.051

**Table 5:** Bivariate analysis of psychosocial health with participants’ illness perception.

Psychosocial Needs Affected	Odds Ratio/(95% Confidence Interval)	*p*-Value (Chi-Squared Test of Independence)
Diet	*	0.004
Hygiene	0.146/(0.047, 0.453)	0.001
Continence	0.271/(0.089, 0.827)	0.011
Self-esteem	0.119/(0.026, 0.546)	0.000
Attractiveness	0.036/(0.005, 0.282)	0.000
Relationships	*	0.000
Intimacy	*	0.000
Leisure/Hobbies	0.063/(0.008, 0.489)	0.000
Clear-mindedness	0.162/(0.035, 0.743)	0.000
Independence	0.102/(0.022, 0.468)	0.000

* Insufficient cell data.
